# Harnessing the Potential of Chimeric Antigen Receptor T-Cell Therapy for the Treatment of T-Cell Malignancies: A Dare or Double Dare?

**DOI:** 10.3390/cells11243971

**Published:** 2022-12-08

**Authors:** Rita Assi, Huda Salman

**Affiliations:** 1 Division of Hematology-Oncology, Stony Brook University, Stony Brook, NY 11794, USA; 2 Division of Hematology-Oncology, Indiana University School of Medicine, Indianapolis, IN 46202, USA

**Keywords:** T-cell neoplasms, CAR-T, target antigen, fratricide, T-cell aplasia, gene editing

## Abstract

Historical standard of care treatments of T-cell malignancies generally entailed the use of cytotoxic and depleting approaches. These strategies are, however, poorly validated and record dismal long-term outcomes. More recently, the introduction and approval of chimeric antigen receptor (CAR)-T cell therapy has revolutionized the therapy of B-cell malignancies. Translating this success to the T-cell compartment has so far proven hazardous, entangled by risks of fratricide, T-cell aplasia, and product contamination by malignant cells. Several strategies have been utilized to overcome these challenges. These include the targeting of a selective cognate antigen exclusive to T-cells or a subset of T-cells, disruption of target antigen expression on CAR-T constructs, use of safety switches, non-viral transduction, and the introduction of allogeneic compounds and gene editing technologies. We herein overview these historical challenges and revisit the opportunities provided as potential solutions. An in-depth understanding of the tumor microenvironment is required to optimally harness the potential of the immune system to treat T-cell malignancies.

## 1. Background on T-Cell Malignancies

### 1.1. Historical Challenges

T-cell malignancies are a heterogeneous spectrum of orphan diseases generally associated with dismal outcomes [1]. They are broadly categorized, according to the maturity level of affected T-cell, into T-cell lymphomas (TCLs) that account for 10% to 15% of all non-Hodgkin lymphomas (NHLs) in adults, and T-cell leukemias (T-ALL) [2]. TCLs could be further subclassified into peripheral TCLs (PTCLs) and cutaneous TCLs (CTCLs) [2,3]. The initial management of T-cell malignancies generally consists of intensive combination chemotherapy, often producing acceptable response rates at the expense of profound toxicity [4,5,6]. The recent introduction of brentuximab vedotin in addition to chemotherapy for the frontline treatment of CD30-expressing PTCLs improved survival outcomes according to the ECHELON2 phase III trial [7]. Nevertheless, these findings are generally pertinent to patients with the anaplastic large cell lymphoma (ALCL) subtype who accounted for 75% of ECHELON2 population; the extended benefit of this combination to CD30+ beyond ALCL remains therefore uncertain. Patients with advanced CTCL derive even lower benefits with initial systemic therapy and the progression-free survival (PFS) improvement for responders is less than 50% [8].

When compared to B-cell malignancies, the use of these frontline strategies has often proved inefficient to maintain remission and subsequent salvage therapy is generally suboptimal, leading to an overall detrimental prognosis [9,10]. Indeed, for the small percentage of eligible relapsed and refractory (R/R) patients with TCLs achieving complete remission (CR) after salvage chemotherapy, allogeneic hematopoietic stem cell transplantation (HSCT) has historically remained the only curative option in 30% of cases [11,12]. The outcomes of patients with chemotherapy R/R T-cell malignancies thus continue to be unsatisfactory in view of the limited availability of effective and well-tolerated therapies.

### 1.2. Immunotherapy in T-Cell Malignancies: About Time

The recent emergence of immunotherapy modalities and their recorded clinical benefit in several hematological malignancies naturally paved the way for their extensive investigation into T-cell neoplasms. Beyond monoclonal antibodies (mAbs), immune checkpoint inhibitors (ICPIs), and bispecific T-cell engagers, the chimeric antigen receptor (CAR)-T cell therapy has particularly presented as a promising strategy for the treatment of several R/R hematologic malignancies [13].

The first CARs converged T-cells to target CD19, a ubiquitous antigen universally expressed on the surface of lymphoid B-cells, generating impressive response rates and leading to the first FDA-approved T-cell therapy for cancer [14,15,16,17]. Beyond CD19, the successful performance of several CAR-T products in treating several R/R B-cell malignancies [17,18] has prompted an expansion of this strategy to different tumors. More specifically, the growing success of CAR-T therapy in B-cell malignancies sparked the development of huge efforts to mirror this breakthrough in T-cell malignancies. As B- and T-cells share several biological architecture and functions, applying CAR T-cell therapy to T-cell neoplasms initially seemed natural until the unfolding of serious practical concerns of fratricide and severe immunosuppression secondary to aplasia of normal T-cells. These findings are further complicated by a strongly protumor immunosuppressive microenvironment that facilitates the development and progression of T-cell malignancies, mostly TCLs [19,20,21].

In this review, we discuss the potential target antigens, as well as the preclinical and clinical efforts invested in adapting CAR-T therapy for T-cell malignancies. We also examine the challenges involved in translating the application of this strategy from bench to bedside. Lastly, we highlight potential novel approaches and proposed solutions to optimize the successful implementation of CAR-T therapy in the realm of T-cell malignancies.

### 1.3. Fundamentals of CAR-T Structure and Mechanisms of Action

CAR constructs are synthetic fusion proteins that endow specific effector cells such as T or natural killer (NK) cells with the property to channel their cytotoxicity toward a designated tumor cell mutually expressing the loaded CAR antigen. Each CAR molecule, therefore, comprises four domains: an antigen recognition domain consisting of an extracellular binding site generally involving a single-chain fragment of the variable region (scFv) of a monoclonal antibody against a specific antigen (e.g., CD19, CD20, etc.); a transmembrane domain and an intracellular signaling domain [22,23]. Nanobodies (also known as VHH) [24] and various toxins have also been used instead of monoclonal antibodies for the same purpose [25]. scFv connects with the transmembrane domain through a hinge/spacer anchor derived from IgG4 or CD8 molecules to initiate signal transduction [26], thereby partially regulating the activity and safety of CAR-T cells. The intracellular pocket subsequently operates as a signaling domain through the CD3ζ chain of the CD3 complex of the T-cell receptor (TCR), and one or two costimulatory domains such as CD28, ICOS, 4-1BB (CD137), or OX40 (CD134). Of note, the presence of costimulatory domains in CAR constructs maintains the activation, proliferation, and persistence of T-cells [27].

CAR transgenes are integrated into T-cells either transiently through mRNA electroporation, or permanently using lentiviral or gammaretroviral gene delivery [28,29].

Unlike normal T-cells that operate through TCR-based ligand recognition, CAR-T cells recognize and eliminate unprocessed tumor antigens independently of the human leukocyte antigen (HLA) complex. This property allows the CAR products to overcome major pathways of tumor escape including lower expression of HLA class I molecules as well as abnormal antigen editing and processing by antigen-presenting cells (APCs) [30].

Once CAR products recognize tumor-specific antigens (TSAs) or tumor-associated antigens (TAAs) on the target cancer cell, the intracellular signaling domains activate the immune effector and memory functions of the CAR-T cells. Accumulating on the surface, CAR-T cells then form an immunological synapse with subsequent activation and proliferation of T-cells, infiltration of tumor sites, cytokines secretion, cytolytic degranulation, the release of perforin and granzyme B, and eventual direct lysis of the tumor cell through several kinetics, dependent on the whether the CAR harbors CD4+ or CD8+ T-cells [31].

While the kinetics of CAR-T operations remain to be fully elucidated, it is imperative that these cells possess the necessary machinery of trafficking and homing to tumor sites, including the hard-to-reach spots, in order to recognize their target antigen and initiate an appropriate cytolytic activity.

### 1.4. Evolution of CARs Design

Since the first conceptualization of engineered T-cells in the late 1980s, CAR manufacturing and design have considerably evolved, mostly over the last few years. CAR T-cell products are currently classified into five generations according to the number of co-stimulatory molecules within the intracellular domain. The first-generation CAR lacked co-stimulatory domains and comprised a CD3ζ chain as an essential carrier of endogenous TCR signals [32]. Despite promising preclinical results and relative success in phase I trials, isolated CD3ζ stimulation was insufficient to mount optimal T-cell signaling, activation, and expansion upon antigen exposure, resulting in impaired antitumor activity [33].

Second-generation CAR-T constructs were subsequently built with a CD3ζ chain and an intracellular signaling domain carrying a co-stimulatory molecule, typically CD28 and 4-1BB (CD137), as well as OX40 (CD134) and induction T-cell stimulator (ICOS, CD278), each with a different effect on T-cells [32,34]. This strategy was evaluated in patients with relapsed B-acute lymphoblastic leukemia, resulting in excellent CR rates [35].

In parallel, third-generation CARs involve two signaling domains along with the CD3ζ chain, such as the CD3ζ -CD28-OX40, leading to more effective anti-tumor responses when compared to second-generation products [36]. While signaling through CD28 generates rapid T-cell activation with loaded cytokine production, aerobic glycolysis, and decreased T-cell persistence, the 4-1BB pathway enhances oxidative metabolism and T-cell persistence despite a slower T-cell response and milder cytokine secretion.

The fourth generation of CARs known as “T-cells redirected for antigen-unrestricted or universal cytokine-initiated killing” or “TRUCK” are actually second-generation CAR-based receptors armored with transgenic payloads such as cytokines and other pro-inflammatory molecules [32,34]. For instance, these CARs can stimulate the secretion of IL-12 that attracts the innate immune cells towards malignant cells, a strategy particularly promising for the treatment of solid tumors where CARs lack the ability to target antigen-negative neoplastic cells [37].

Finally, the fifth generation of CARs shares vast similarities with the fourth generation with the exception of an intracellular domain harboring a cytokine receptor instead of a cytokine expression stimulator [38]. These constructs facilitate a target antigen-dependent activation of the JAK-STAT pathway that further amplifies their proliferation while blocking terminal differentiation [38].

It is important to note that all commercially available CARs present several limitations as they belong to the second generation, they comprise genetically modified patient-derived (autologous) peripheral blood T cells and use viral vectors for the delivery of CAR transgenes into T-cells [39].

### 1.5. Challenges to CAR T-Cell Therapy in T-Cell Malignancies (Figure 1)

As the downstream signaling pathways inducing T-cell activation operate independently of the MHC complex, any surface antigen whose expression is confined to neoplastic rather than normal cells could therefore present as a potential target for CAR-T therapy [40]. The mainstay of developing any safe and effective CAR-T cell therapy is the identification of an ideal surface target antigen that is highly sensitive for the underlying malignancy and uniformly specific to circumvent on-target off-tumor toxicities. In general, hematological malignancies represent a heterogeneous population where an optimal antigen, theoretically expressed on all malignant cells with a robust intensity, rarely exists.

The development of CAR-T therapy against T-cell neoplasms remains specifically problematic when compared to that in the B-cell compartment. Resetting T-cells to eliminate malignant T-cells while sparing normal T-cells is a complicated task. The major obstacle stems from the limited availability of T-cell malignancy-specific target antigens to construct a CAR molecule. Indeed, the majority of antigens (such as CD3, CD5, and CD7) targeted by CAR-T products against T-cell malignancies are readily expressed by normal T cells [41,42,43]. This overlapping expression complicates the isolation of healthy T-cells from patients with T-cell malignancies to engineer autologous CAR-T products where normal and malignant T-cells are jointly recovered during leukapheresis. In this situation, the autologous CAR-T product possibly incorporates T-cells generated from malignant T lymphocytes. This “impurity” subsequently generates CAR-T-mediated ablation of normal T-cells after product infusion, a potentially fatal phenomenon of profound immunodeficiency known as T-cell aplasia [44]. Finally, a CAR-T construct targeting a TAA jointly expressed by different populations of T-cells can blindly intercept malignant T-cells, normal T-cells, and other CAR T-cells [45]. When the latter express a target antigen on their surface, a mechanism of fratricide develops during manufacturing whereby CAR-T cells attack and annihilate each other, eventually disrupting their in vivo expansion, persistence, and tumoricidal function [46].

All of the aforementioned obstacles have greatly hindered the development of successful CAR-T therapy for the treatment of T-cell malignancies. As seen in B-cell malignancies, therapeutic failure of CAR-T therapy in T-cell malignancies also seems to sequester around two major patterns: inherent resistance signaling the absence of significant disease response after therapy or acquired resistance in which patients enjoy a transient response followed by disease recurrence. All models of CAR-T failure appear to stem from tumor-intrinsic evasion mechanisms that are either antigen-dependent (loss of antigen expression, fratricide, etc.), or independent. Additionally, pre- and/or post-exposure T cell dysfunction could occur, leading to resistance to CAR-T therapy and/or disease relapse. Based on all these observations, several groups have subsequently devised various products and strategies, including the targeting of more restricted T-cell antigens such as CD4, CD30, CD37, and CCR4 (Table 1). All antigens, however, present variably even within the same type of T-cell malignancies. Therefore, choosing the right target and considering the potential for adverse events is an area of unmet need in CAR-T therapy for T-cell malignancies.

Other alternative antigens, such as the myeloid markers CD13 and CD33, have also emerged as potential targets owing to their aberrant expression on the surface of precursor T-cell leukemia that possibly portends a worse disease prognosis [47]. It is important to note that despite these potential solutions, the development of CAR-T cell therapy remains burdened by a set of serious adverse events, such as cytokine release syndrome (CRS), neurotoxicity, off-target toxicity, as well as high costs.

## 2. Addressing Fratricide

### 2.1. Targeting Pan-T Antigens

#### 2.1.1. CD3

CD3 is a historically favorable target for the use of immunotherapy in T-cell malignancies owing to its restricted expression to the hematopoietic compartment, specifically mature T-cells. CD3 forms a complex with the TCR, thereby stimulating target antigen recognition with subsequent T-cell activation [42]. CD3-based immunotherapy, in the form of mAbs was extensively evaluated for the treatment of several T-cell malignancies with marginal clinical efficacy [48,49]. The subsequent use of CD3 as a target for CAR-T therapy was largely hindered by fratricide, [50,51] secondary to the overlapping expression of CD3 on the normal and malignant T-cells. These observations generated the need for fratricide-resistant products or incorporating different effector cells that lack the expression of the target antigen.

A proposed solution entailed the design of a novel third-generation CD3-based CAR embedded in the NK-92 cell line [52]. Of note, NK cells lack CD3 expression, possess shorter lifecycles when compared to T-cells, and express the IgG Fc fragment. The latter is a low affinity less proinflammatory III receptor (FcRYIII) that endows NK cells with the potential to execute antibody-dependent cell-mediated cytotoxicity without risk of graft versus host disease (GvHD) [52,53,54]. Chen et al. demonstrated that the CD3CAR transduced NK-92 cells possess significant dose-dependent cytotoxic activity in vitro, as well as an in vivo effects against CD3-expressing PTCL samples and several T-acute lymphoblastic leukemia/lymphoma (T-ALL) cell lines, as well as prolonged survival in preclinical models engrafted with the Jurkat cell line [50]. Furthermore, the study established the specific potential of CD3CAR NK-92 cells to target CD3 antigen without off-target effects. These preclinical data could pilot the investigation of CD3CAR modified NK cells for the treatment of CD3^+^ T-cell malignancies, thereby serving as a bridge to HSCT or other definitive therapies.

More recently, gene-editing technologies, such as the transcription activator-like effector nuclease (TALEN), have been increasingly utilized to disrupt the CD3/TCR complex and prohibit the endogenous TCR expression on T-cells, before manipulating these cells to express the CD3ε-targeting CARs [51]. Such a method produced specific and significant tumoricidal activity against pediatric samples of T-ALL, as demonstrated in preclinical models engrafted with the CD3+ Jurkat cell line [51].

#### 2.1.2. CD5

CD5 is a membrane-based glycoprotein with an extracellular domain normally restricted to thymocytes, peripheral T cells, and a subset of B lymphocytes known as B-1a cells [41]. This antigen is also commonly expressed in several T-cell malignancies such as T-ALL and PTCL [55,56]. CD5 promotes the survival of lymphocytes while negatively regulating TCR signaling pathway [57,58], and has therefore been historically considered a suitable target for use in immunotherapeutic strategies [59].

Earlier studies by Mamonkin et al. of CD5CAR-T therapy against T-ALL and TCL samples showed complete antitumor response in vitro, but only limited tumoricidal activity in animal models [45]. Disease recurrence was attributed to a phenomenon of partial and transient fratricide (primarily against naïve and central memory cells) that subsided 3 days post-administration.

To overcome this limitation, Chen and colleagues incorporated NK-92 cell lines in the design of a third-generation CD5-redirected CAR, as NKs lack CD5 expression on their surface [60]. Preclinical data showed a steady expansion of the composite product ex vivo, with selective and significant tumoricidal activity towards several T cell lines including Jurkat, CCRF-CEM, and MOLT-4, as well as against primary CD5+ cells of human PTCL and T-ALL samples [60]. More specifically, mouse xenograft models of T-ALL demonstrated improved survival as well as substantial reduction in their tumoral mass [60].

Similar results were replicated by using CRISPR-Cas9 technology to knockout CD5 in T cells before embedding the CAR transgene into Jurkat and primary patients’ cells. This experiment by Raikar et al. resulted in limited fratricide and subsequent CAR persistence [61]. Mamonkin and coworkers also reported that substituting the 4-1BB co-stimulatory domain for the original CD28 in the endodomain of CD5CARs can enhance the differentiation capacity of the CAR compound, resulting in increased antitumor efficacy [62]. To avoid fratricide and target antigen exhaustion, a Tet-Off system capable of interfering with CAR transduction was implemented in the presence of doxycycline ex vivo, sustaining the recovery and expansion of CAR surface expression in vivo in the absence of immediate fratricide [62]. A more recent study comparing the use of the 2B4 and 4-1BB costimulatory domains in CAR-NK compounds found a similar in vitro selective tumoricidal activity [63]. Notably, CAR-NKs harboring the 2B4 co-stimulatory domain further demonstrated an improved antileukemic activity in xenograft T-ALL preclinical models [63].

In a phase I clinical trial (NCT03081910; MAGENTA trial) of 14 patients with R/R TCL patients, autologous second-generation CD28-costimulated CD5CAR-T therapy resulted in sufficiently durable tumoricidal responses that enabled a transition to HSCT without significant T-cell aplasia or high-grade CRS [64]. It is noteworthy that responses did not correlate with infused doses or level of T-cell expansion, findings attributed to a shortened manufacturing with cryopreservation for 3–5 days post-transduction instead of a standard 7-day expansion [64].

#### 2.1.3. CD7

CD7 is a glycoprotein member of the Ig superfamily that is normally expressed on T and NK cells [65] and aberrantly on T-ALLs and TCLs [55], subsequently posing a risk of fratricide when used as target for of CAR-T cell therapy. Gomes-Silva et al. therefore adopted CRISPR-Cas9 genome editing to disrupt the CD7 expression before its manipulation for CAR integration [43]. This approach resulted in enhanced CAR expansion, strong and selective tumoricidal activity against several CD7-expressing cell lines (Jurkat, CCRF, MOLT-4, Sup-T1, and Hut78), as well as against human T-ALL samples and xenograft models with variable levels of CD7 expression [43].

CRISPR-Cas9 was also utilized to engineer CD7-redirected CAR-T products lacking CD7 and TCR alpha chain (TRAC) expression [46]. This fratricide-resistant construct demonstrated significant antitumor activity against T-ALL cell lines and primary human samples, as well as tumor regression in preclinical models with the absence of GvHD [46]. Strategies involving gene-editing tools might prove useful for the production of allogeneic CAR-T models while minimizing donor-derived T cells’ alloreactivity before the use of off-shelf products. Such an approach was adopted in the design of the universal CD19-targeted CAR-T cell product for the treatment of B-NHL [66,67], whereby healthy donors derived allogeneic CAR-T cells can substitute patient-derived inefficient T cells and reduce the cost and time of production.

Blockade of CD7 expression in T cells is another suggested approach to mitigate fratricide. Png et al. designed a protein expression blocker (PEBL) system composed of a CD7-targeting scFv fused to a retention domain that intercepts CD7 in the ER/Golgi, thereby blocking its normal expression [68]. This strategy prevented fratricide while preserving CARs expansion and their tumoricidal activity, as well as INF-γ and TNF-α response. More importantly, such CARs exhibited pronounced antileukemic activity against several CD7-expressing cell lines and patient-derived xenografts models of T-ALL [68].

Owing to their aforementioned properties, NK cells have also been manipulated for the production of CAR-T cells. You et al. designed monovalent and bivalent composite constructs using NK-92MI cells and CD7-specific nanobodies as the targeting domain [69]. While both CAR-NKs products showed selective and effective tumoricidal activity towards cell lines and tumor samples of T-ALL, the bivalent model scored superior antitumor effects and generated higher levels of granzyme B and IFN-γ. These results were subsequently reproduced in preclinical assessments of xenograft models of T-ALL [69]. Early results of the first-in-human clinical trial (NCT04004637) utilizing allogeneic CD7NKCAR-Ts in patients with CD7+R/R T-ALL or T-cell lymphoblastic lymphoma pre- or post-HSCT were recently reported [70]. The study showed CR at 30 days in 18 out of 20 (90%) patients, allowing 37% of responders to proceed to HSCT. Of the 12 patients who did not receive HSCT, 9 remain in remission at a median follow-up of 6.3 months. Normal T and NK cell aplasia were seen, leading to opportunistic infections in some patients. The majority of these patients had, however, recovery of small numbers of CD7– peripheral T and NK cells. Other clinical trials (NCT04033302 and NCT03690011) are currently evaluating the feasibility and efficacy of this target antigen for the treatment of several T-cell malignancies (Table 1).

### 2.2. Targeting Antigens with Restricted Expression

#### 2.2.1. CD1a

CD1a is expressed on the surface of developing cortical thymocytes and T-ALL cells, and absent on T cells and CD34+ progenitor hematopoietic cells [71,72]. This characteristic pattern of expression allowed CD1a to present as a fratricide-resistant target for CAR-T therapy with robust tumoricidal activity and considerable persistence against CD1a-expressing T-ALL cell lines and primary cells of cortical T-ALL samples [73]. Moreover, CD1aCAR-Ts offer the potential for minimal on-target off-tumor toxicities. Further clinical assessments are needed to establish the real suitability of CD1a for CAR-T therapy of cortical T-ALL patients.

#### 2.2.2. CD4

CD4-based immunotherapy in the form of mAbs has been extensively investigated for the treatment of TCLs, with acceptable clinical activity, safety profile, and low immunogenicity [74,75,76,77], making CD4 an attractive target for use in CAR-T therapy. Early preclinical data of a third-generation CD8+CD4CAR-T construct demonstrated the selective activity of the construct against CD4-expressing cell line (KARPAS 299 cells) and patient-derived PTCL samples while conserving their memory stem cell-like phenotype [78]. Furthermore, the experiment showed that the CD4-manipulated CARs possessed antitumor activity in mouse models, leading to prolonged survival when compared to the control GFP-expressing T-cells. To avoid the risk of T-cell aplasia and related opportunistic infections, another third-generation CD4-redirected CAR-NK was engineered using the NK-92 cell line [79], owing to its shortened persistence and lower risk of GvHD, as detailed above. This construct exhibited a dose-dependent selective tumoricidal capacity against several aggressive CD4-expressing patient-derived cell samples and cell lines of T-ALL and TCLs, with positive effects on the survival of the preclinical xenograft models [78,79]. Altogether, these robust preclinical data strongly support the potential role of CD4CAR NK cells as a conditioning regimen bridging to definitive HSCT, or as a possibly self-sufficient curative option for certain patients with T-cell malignancies.

Despite the use of NK cells, CD4CAR T-cells remain a problematic approach as the inadvertent massive eradication of normal CD4+ T-cells leads to T-cell aplasia and an HIV/AIDS-like syndrome [80]. If this phenomenon is anticipated to be profound and irreversible, it necessitates the use of a physiologic “safety switch” that aborts the heightened activity of CD4CARs post-administration and achievement of tumoricidal activity. Ma et al. incorporated the CD52-specific humanized mAb alemtuzumab for this purpose since CD52 is expressed on the surface of both normal and malignant lymphocytes [81]. Alemtuzumab achieved >95% depletion in the number of circulating CD4CAR-T at 6 and 48 h after its administration, suggesting its potential role in hindering unwanted toxicities [81]. An ongoing clinical trial is currently evaluating the clinical safety and efficacy of a third-generation CD4CAR-T for the treatment of T-cell malignancies (NCT03829540) (Table 1).

#### 2.2.3. CD30

CD30, or TNFRSF8, is a member of the TNF receptor superfamily and is expressed by small subsets of normal B and T cells, as well as in several malignancies including HL, ALCL, ATLL, and PTCL [82,83,84]. CD30 expression is thought to increase following administration of chemotherapy in T-ALL patients, hence its potential benefit for targeting in R/R cases [85].

The successful immunotherapeutic targeting of CD30 was established through brentuximab vedotin, an anti-CD30 antibody–drug conjugate (ADC) that recorded marked clinical benefits in patients with HL and some TCL subtypes [86,87]. Additionally, it has been previously shown that M2 CD163^+^ macrophages heavily express CD30, suggesting that the effects of brentuximab vedotin may be at least partially explained by architectural modifications in the tumor microenvironment, hence the anecdotal reports of the ADC’s activity in CD30- lymphoma [88]. However, several constraints of antibody-based therapy such as limited tumor penetration and antigen-mediated clearance with subsequently shortened response duration prompted the exploration of the CAR pathway for a more efficient CD30 targeting [89]. Studies assessing the feasibility of such an approach date back to more than 20 years [90,91] while early clinical data were recently reported from a phase I dose-escalation clinical trial (NCT01316146) of 7 R/R HL and 2 ALCL patients who received second-generation CD30CAR-Ts with no recorded toxicities [92]. Two of the seven HL patients achieved CR lasting more than 2 years and another three patients had transient stable disease. Nevertheless, CR was seen in only one of the two ALCLs and lasted 9 months [92]. Several other studies involving CD30CAR-T were conducted exclusively in HL patients, suggesting that in vivo expansion of the compound was dose-dependent and that fludarabine-based preconditioning therapy produces durable responses with an acceptable safety profile [93,94]. It is important to note that the overall results seen with this strategy were suboptimal as most patients required multiple CD30CAR-T injections to achieve a stable disease status. Additionally, extra-nodal lesions appeared to respond less than the nodal compartment and T-cells showed short persistence of around two months following infusion [92,95,96].

Guercio et al. subsequently attempted at improving the antitumor activity and homing capacity of CD30CARs through a third-generation design incorporating a combination of OX40 and CD28 costimulatory molecules, and the production machinery of IL-7 and IL-15 [97]. This construct generated prolonged persistence with increased proliferation of T-cells, along with sustained immunology against lymphomatous cells [97].

CD30 remains an important target that deserves further exploration. Ongoing clinical trials (NCT03049449, NCT02917083, NCT02663297, NTC01316146, NTC03602157, NTC0227458) will potentially clarify its role in CAR-T cell therapy for T-cell malignancies (Table 1).

#### 2.2.4. CCR4

C–C chemokine receptor type 4 (CCR4), also known as CD194, is expressed by several normal T-cell populations including regulatory T-cells (Tregs), Th2, and Th17 cells, and overexpressed on malignant T-cell subsets of patients with PTCL, CTCL and ATLL [98,99]. CCR4 was previously targeted using the first-in-class humanized mAb Mogamulizumab that is currently approved for R/R CTCLs, with limited clinical responses [100]. Perera et al. subsequently demonstrated that allogeneic CCR4CAR-Ts achieved significant tumoricidal activity against CCR4-expressing patient-derived tumor cell lines and xenograft models of ATLL [101]. These positive findings were, however, hindered by undesirable skin toxicities such as Stevens–Johnson syndrome attributed to the expression of CCR4 on normal T-cell populations, similar to what was previously reported with the use of mogamulizumab [102,103].

Di Stasi et al. previously showed that CD30CAR-Ts armored with CCR4 as a cognate receptor for CCL17 possess improved tumor homing and tumoricidal activity compared with CD30CAR-Ts that lack CCR4 [95]. Since CD30 expression is also retained in R/R lymphoma, it is thought that a bicistronic CCR4CD30CAR-T product would be more potent mostly in CD30+ CTCL due to enhanced trafficking to the skin. Preliminary results of a clinical trial (NCT03602157) investigating the safety and activity of this approach in 10 R/R HL and CD30+ CTCLs previously treated with brentuximab vedotin showed that 75% of HL patients achieved CR while 1 of 2 CTCL patients achieved stable disease as best response [104]. While still suboptimal for T-cell malignancy patients, the trial provides a proof of concept on the feasibility and safety of this strategy to improve homing of CAR constructs.

#### 2.2.5. CCR9

C–C chemokine receptor type 9 (CCR9), or CD199, is a seven-pass transmembrane G–coupled receptor for CCL25. CCR9 is involved in early T-cells development and migration [105]. It is expressed in gut γδ intraepithelial T-cells in mice and heavily on R/R T-ALL, on less than 5% of normal circulating T and B cells [106]. As human and murine CCR9 share around 86% of homology in their sequences, these properties make CCR9 a viable target for CAR-T therapy in T-ALL. Maciocia et al. constructed a second-generation CAR, incorporating CD8 stalk/transmembrane domain and 4-1BB-CD3zeta endodomain and using RQR8 as a suicide switch [107,108]. These CCR9CAR-T cells demonstrated significant tumoricidal activity against several in vitro and in vivo models of T-ALL, without fratricide or lysis of normal T-cells [107]. These findings warrant further exploration of this product, mostly for ETP-ALL patients, a high-risk subset with unmet needs [109].

#### 2.2.6. CD37

CD37 is a leukocyte-exclusive antigen expressed on the surface of mature B-cells that regulates T-cell proliferation at different levels [110,111,112]. CD37 is also detected in several T-cell malignancies and the feasibility and safety of its targeting were previously investigated via an ADC named AGS67E, mostly in CTCL (NCT02175433) [113]. A CD37CAR was subsequently reported to mount an in vitro target antigen-dependent tumoricidal activity against TCLs with variable levels of CD37 expression in the absence of a significant fratricide [114]. Based on these observations, baseline screening for the expression of CD37 might be needed before considering patients with T-cell malignancies for preclinical and clinical testing with this CAR.

#### 2.2.7. TRBC1 and TRBC2

The TCR plays a major role in normal T-cell proliferation through the recognition of antigens presented by APCs. TCR possesses an alpha chain and a beta chain with the constant region of the latter being expressed either through the T-cell receptor beta constant 1 (TRBC1) gene or the T-cell receptor beta constant 2 (TRBC2) gene [115]. While malignant T-cells significantly downregulate TCR, it remains expressed by around 30% of T-ALLs and the vast majority of PTCLs [116]. Based on the fact that a normal population of T cells expresses both TRBC1 and TRBC2 and a malignant subset expresses either one, targeting either receptor at a time could have antitumor activity while preserving a significant proportion of normal T-cells.

For this purpose, Maciocia et al. manufactured TRBC1CAR-Ts and showed that the construct spares TRBC2+ cells, in vitro [117]. Furthermore, those treated with these cells exhibited a substantial reduction in tumor burden and prolonged survival when compared to the control group [117].

In another study, gene editing technologies were used to simultaneously delete the expression of one of the TRBC genes [118]. This disruption led to the abolition of endogenous TCR from the cell surface, concluding that such a strategy could be reserved to prevent fratricide when producing autologous TRBCCAR-Ts [118]. An ongoing phase I/II clinical trial (NCT03590574) is currently evaluating the safety and efficacy of *AUTO4,* a TRBC1CAR-T therapy for patients with TRBC1^+^ TCLs including PTCL, ALCL, and angioimmunoblastic T-cell lymphoma (AITL).

## 3. Addressing T-Cell Aplasia

T-cell aplasia results from on-target off-tumor effects of CAR-T therapy against normal T-cells that mutually express the CAR-specific target antigen [119]. T-cell aplasia is serious toxicity owing to an increased risk of life-threatening infections that largely hinder the successful implementation of CAR-T therapy for patients with T-cell malignancies [44].

Several strategies have been investigated to prevent T-cell aplasia, including the use of target antigens largely restricted to malignant cells, as detailed above. Additionally, selective targeting of an antigen exclusively expressed in a subset of T cells could allow the intact T-cell population to develop sufficient immunity during CAR-T therapy. This strategy, adopted by Maciocia et al. who utilized TRBC1 or TRBC2 as CAR-T targets, aborted full fratricide as well as t-cell aplasia [117].

Another approach to mitigate T-cell aplasia entails the use of CAR-T constructs with controllable or limited longevity and tumoricidal activity. In particular, CAR-T products engineered using viral vector transduction generate robust expansion and persistence in vivo, leading to heightened risks of T-cell aplasia [120], while mRNA engineered CAR-T cells have shown similar tumoricidal activity with limited persistence following administration [121,122]. These results were replicated by two pilot clinical trials of R/R HL patients (NCT02277522 and NCT02624258) treated with non-viral mRNA-electroporated CD19CAR-T [120]. This approach could potentially be beneficial for patients with T-cell malignancies. However, either sequential CAR-T administration or bridging to HSCT might be needed to achieve stable and sufficient tumoricidal activity.

Equipping CAR-T products with safety switches (or suicide switches) that allow for control of transduced T-cells after injection into patients has also been suggested to limit T-cell aplasia [123]. Several forms of safety switches exist with different applicability, advantages, and inconveniences. For instance, metabolic switches, such as those transduced with herpes simplex virus thymidine kinase, can be hampered by potential immunogenicity [124], unlike the inducible human caspase (iCasp) switches [125]. The exclusive eradication of the adoptively transferred T-cells could be also performed through a specific mAb that concomitantly blocks the same antigen targeted by CAR-T constructs [126]. Nevertheless, this method predisposes individuals to undesirable adverse events caused by the inadvertent targeting of normal tissues that could simultaneously express a particular target antigen. Several studies (NCT02028455, NCT03016377, NCT01815749) evaluating the feasibility and efficacy of suicide switches are currently ongoing.

### 3.1. CAR-T Product Contamination with Malignant T Cells

The generation of autologous CAR-T cells for the treatment of B-cell malignancies carries a minimal risk of contamination by malignant cells whereby the target antigen becomes unrecognizable by the CAR product, leading to therapy failure [127]. In parallel, manufacturing autologous CAR-Ts from patients with T-cell malignancies portends a higher incidence of contamination during T-cell isolation due to antigen similarity between the normal and malignant compartments. While the use of a healthy donor to produce allogeneic CAR-Ts appears as a potential solution to avert contamination and possibly T-cell aplasia, these products possess a shorter in vivo persistence and involve a risk of GvHD or mass eradication by the recipient’s immune system [128].

As previously described, a very common approach has been the use of NK cells as effector cells owing to the lack of expression of certain TAAs seen on normal and malignant T-cells, their shorter longevity, and lower proinflammatory properties compared to T-cells [50,60]. CAR-modified NK cells would therefore be eliminated shortly after administration, thereby decreasing the risk of fratricide, T-cell aplasia, and GvHD in case of use of allogeneic products, and potentially removing the need for an inducible safety switch [129]. The challenges of in vitro expansion of NK cells and the CAR transduction into them are generally resolved by using the NK92 cell line as an alternative [130,131,132]. Nevertheless, serious concerns about the potential tumorigenicity of NK cell lines remain, despite the use of NK-92. For this purpose, these cell lines undergo treatment with radiation before administration to patients, a measure that increases their safety but significantly reduces their cytotoxicity [131]. The optimal irradiation dose used in this strategy remains under investigation. Finally, several studies reported the expiration of PDX models during in vivo assessment within minutes of CD3 and CD5CARNK therapy secondary to strokes induced by NK cell aggregation [50,60].

These observations strongly suggest the need for more in-depth preclinical information to conclude on the safety and feasibility of NK cell lines as allogeneic effectors for CART-cell therapy.

Cell and gene editing technologies are being increasingly utilized to develop off-the-shelf allogeneic CAR-T constructs that are resistant to fratricide without incurring a risk of GvHD. These include CRISPR-Cas9 used to develop off-the-shelf CD7CAR-T by knocking out TRAC in T cells before viral transduction [46], as well as TALEN and Zinc-finger nucleases (ZFN). Preclinical data on the feasibility of these tools have been rather investigated in hematological malignancies other than T-cell neoplasms [133].

Another approach to avoid contamination and its subsequent risks entails the use of multi-virus-specific T (VST) cells as effector cells for CAR expression. These genetically engineered cells usually lack the expression of the CAR target antigen, and are, hence fratricide resistant. Furthermore, they offer the potential for antiviral activity in the event of T-cell aplasia [134,135]. Based on these findings and the study by Melenhorst et al. suggesting that allogeneic VST cells with HLA alloreactivity do not induce GvHD in humans [136], such T cells may provide a real opportunity for producing off-the-shelf CAR-Ts.

In addition to fratricide, T-cell aplasia, and GvHD, CAR-T cells have been hampered at times by the inability to achieve optimal trafficking to difficult tumor sites, including the skin. This is likely attributed to the fact that αβ T cell subsets can poorly infiltrate such locations. γδ T cells, a smaller population accounting for only 1–5% of circulating lymphocytes, are ubiquitous in the skin, intestine, and reproductive apparel and could express chemokine receptors attracting them to home in inaccessible tumor location [137,138,139]. Furthermore, γδ T cells could extensively proliferate ex vivo and do not induce GvHD as activation of their TCR is MHC-independent [140]. A specific variant of γδ T cells, Vγ9Vδ2, possesses the potential for tumor killing through recognition of certain phosphoantigens such as isopentenyl pyrophosphate, readily accumulated in tumor cells [141,142]. These aforementioned properties enable γδ T cells to present as potential alternative effectors for allogeneic CAR-T therapy in T-cell malignancies, following serial evaluation in studies for various other malignancies [140,143,144].

### 3.2. Combining ICPIs and CAR-T Products in T-Cell Malignancies: An Ongoing Dilemma

The revolutionary success of ICPIs in several solid and hematologic malignancies prompted a major interest in evaluating their application in T-cell malignancies. The development of immunotherapy in these tumors was generally slower and burdened by an imbalance of exhausted malignant T-cells and stimulation of a T-cell response. Furthermore, there exist serious concerns of potential tumor hyperprogression induced by the blockade of inhibitory signals on the surface of the malignant T-cells, thereby perpetuating the proliferation of these cells [145]. The data regarding the therapeutic utility of programmed cell death protein 1 (PD-1) targeting remains controversial, especially with the discovery of its haploinsufficient tumor suppressor function in preclinical TCL models [146].

Prolonged or repeated exposure to CAR-T cells produces T-cell exhaustion with subsequent upregulation of several ICPs such as CTLA4, PD-1, CD160, CD244, TIM3, TIGIT, and LAG-3 [147]. PD-1 antibody therapy is therefore thought to synergize with CAR-T cells to avert T-cell senescence and heighten antilymphoma effects and several others, and the combination is being investigated in B-cell malignancies [147]. Mirroring this strategy in TCLs is hindered by concerns of ICPI-induced hyperproliferation of malignant T-cells and/or CAR-T cells, and clinical trials evaluating the combination are still lacking.

## 4. Perspective

With the expansion of CAR-T therapy, the treatment of T-cell malignancies is not a neglected field anymore. Earlier results of CD5 and CD7 CAR-Ts, and more recently CCR9CART-s, represent a large-scale milestone in the treatment of T-cell malignancies and inspiration to advance this work into further clinical investigation and future application. Several other trials, some with more sophisticated CAR constructs, are currently ongoing and their results are eagerly awaited. Generalization and validation of these results will ultimately need larger studies that should be anticipated in the foreseeable future. However, several interim questions remain unanswered regarding the optimal therapeutic construct to achieve (off-the-shelf versus autologous), the ideal application of gene editing tools, and the solutions proposed to avert CAR-T-related toxicities. With the substantial strides of preclinical data on the tumor microenvironment and immune landscape of T-cell malignancies, the improved knowledge of these diseases will ultimately identify patients most likely to harness best the power of CAR-T therapy.

## Figures and Tables

**Figure 1:**
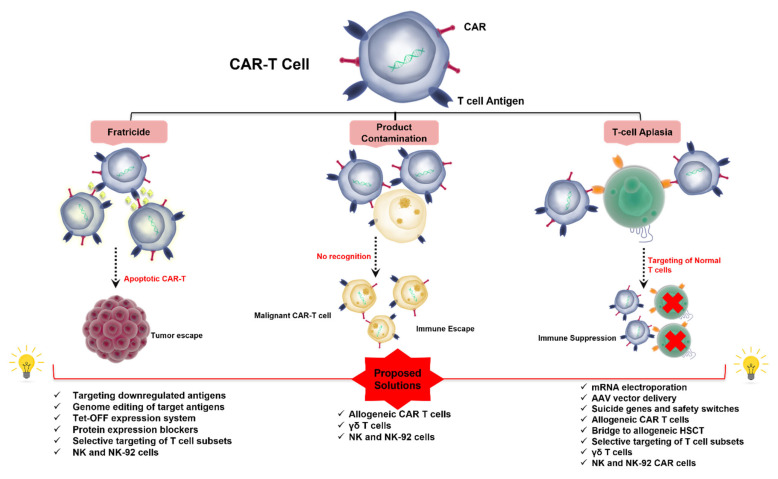
Challenges to the application of CAR-based therapy in T-cell malignancies and proposed solutions. CAR-T cell therapy can be complicated by fratricide, contamination of the final product by transduced tumor cells, as well as T-cell aplasia. CAR: chimeric antigen receptor; AAV: adeno-associated virus; HSCT: hematopoietic stem cell transplantation.

**Table 1:** Available target antigens with their corresponding ongoing clinical trials for CAR-T therapy of T-cell malignancies.

Target Receptor	Notes on CAR Construct	Target Disease	Main Eligibility Criteria	Phase	Target Accrual	Outcomes	Age Group (Years)	Status	ClinicalTrials.gov Identifier
***CD4***									
	Anti-CD4 CAR transduced with a lentiviral vector	R/R CD4+ T-NHL	After failure of standard therapy	I	12	Toxicity DFS; PFS; OS	≥18	Recruiting	NCT04162340
	Autologous LCAR-T2C	T-ALL T-NHL	PD or SD after ≥1 prior line of therapy	I	33	DLT, AE, RP2D, PK	18–75	Recruiting	NCT04973527
	Autologous LCAR-T2C	R/R CD4+ T-NHL	PD or SD after ≥1 prior line of therapy	I	32	DLT, AE, RP2D, PK	18–75	Recruiting	NCT04219319
	Autologous T cells transduced with a lentiviral vector	R/R CD4+ T-NHL	R/R including ASCT	I	20	Safety, feasibility In vivo survival, clinical response	≥18	Active, not recruiting	NCT03829540
	LB1901- autologous CD4-targeted CAR-T	T-NHL	Failed ≥2 prior lines of therapy	I (FIH)	50	RP2D ORR, TTR	≥18	Active, not recruiting	NCT04712864
***CD5***									
	Anti-CD5 CAR transduced with a lentiviral vector	R/R T-ALL and T-NHL	After failure of standard therapy	I	20	Toxicity DFS; PFS; OS	≥8	Recruiting	NCT04594135
	Donor-derived CD5 CAR T cells	R/R T-ALL	After failure of standard therapy	I (FIH)	18	DLT, AEs ORR, BOR	1–70	Recruiting	NCT05032599
	Autologous CD5.CAR/28zeta CAR T cells Allogeneic CD5.CAR/28zeta CAR T cells	R/R T-ALL and T-NHL	Suitable for ASCT R/R post ASCT	I	42	DLT, ORR	Up to 75	Active Not recruiting	NCT03081910 MAGENTA
	Endogenous CD5 in CT125A cells knocked out by CRISPR/Cas9 genome editing	PTCL CD5+ R/R	Failed at ≥1 prior therapy	I	18	DLT, AE ORR, CR	18–70	Not yet recruiting	NCT04767308
***CD7***									
	BT-007, transduced with 4-1BB/CD3ζ lentiviral vector, expanded in vitro	CD7+ R/R T-NHL	Failed ≥1 line of therapy	I	15	ORR, retention time and amount of CAR-T cells remaining in vivo	18–70	Recruiting	NCT05554575
***CD30***									
	Carmelizumab + CD30 CAR-T cells	CD30+ R/R T-NHL	Failed ≥2 lines of systemic treatment	II	30	ORR, OS, DOR, PFS, AEs	18–70	Recruiting	NCT05320081
	Allogeneic CD30.CAR-EBVSTs	CD30+ R/R T-NHL	ALK+/ALK- ALCL PTCL	I	18	DLT, ORR, DOR, SD, PFS	12–75	Recruiting	NCT04288726
	CD30.CAR-EBVST cells	CD30+ R/RT-NHL	ALK+/ALK- ALCL PTCL	I	18	DLT, ORR, DOR, SD, PFS	12–75	Not yet recruiting	NCT04952584
	Autologous 3rd generation anti-CD30 CAR T cells.	CD30+ R/R T-NHL	ATLL, ALCL, AITL, NK/TCL, PTCL	I	50	AEs, OS, EFS, RFS, retention amount	18–70	Recruiting	NCT04008394
	Autologous, uses retroviral vector	CD30+ R/R T-NHL	PTCL, AITL, NK/TCL, ALCL, etc.	I	66	DLT, ORR	12–75	Recruiting	NCT02917083 RELY-30
	Autologous CAR.CD30 T CELLS	CD30+ R/R T-NHL	PTCL, AITL, NK/TCL, ALCL, etc.	I	9	MTD, AE, ORR, DCR, DOR, PFS, OS	18–70	Not yet recruiting	NCT05208853
	CD30-Directed Genetically Modified Autologous T-Cells	CD30+ R/R T-NHL	PTCL, ALCL, NK/TCL	I	21	DLT, ORR, DOR, PFS	18–75	Active, not recruiting	NCT04526834
	HSP-CAR30; Autologous second generation (4-1BBz)	CD30+ R/R T-NHL	ALK+/ALK- ALCL, PTCL	I/II	30	Safety, toxicity, MTD, CR	18–70	Recruiting	NCT04653649
	Autologous CAR.CD30 T CELLS	CD30+ T-NHL	Relapsing after ASCT, or refractory to 2 multidrug regimens and/or anti-CD30 antibody treatment. Newly diagnosed patients unable to receive or complete standard chemotherapy	I/II	30	AE, anti-tumor responses, in vivo existence	16–80	Recruiting	NCT02259556
	Autologous activated T lymphocytes (ATLs) expressing CD30 CAR	CD30+ R/R T-NHL	PTCL	II	20	PFS, BOR, ORR, DLT, OS	18–99	Recruiting	NCT04083495
	Autologous CAR.CD30 T CELLS	CD30+ R/R T-NHL	Failed >2 prior regimens. Subjects relapsed after ASCT or APSCT also eligible	Ib/II	40	2-y OS, 2-y PFS, ORR, DOR, AEs	≥3	Recruiting	NCT02690545
	Transduced with lentivirus bearing anti-CD30 antibody scFV and the activation signals of second-generation CART designation.	CD30+ R/R T-NHL	ALK+/ALK- ALCL, PTCL	I	20	Safety, anti-tumor efficacy	2–80	Recruiting	NCT03383965
	Autologous CAR.CD30 EBV specific-CTLs	Newly diagnosed and R/R T-NHL	CD30+; include failure post APSCT	I	18	Survival and anti-tumor effects in vivo	All ages	Active, not recruiting	NCT01192464 CARCD30
	Autologous CAR.CD30 T CELLS	CD30+ T-NHL	CD30+; include failure post APSCT	I	10	Safety; Survival and anti-tumor effects in vivo	All ages	Active, not recruiting	NCT01316146 CART CD30
	Autologous CAR.CD30 T CELLS with CCR4 (ATLCAR.CD30.CCR4)	CD30+ CTCL	All cutaneous CD30+ T-NHL	I	59	AE, PFS, BOR, ORR, OS	≥18	Recruiting	NCT03602157
	Autologous CAR.CD30 T CELLS with CCR4 (ATLCAR.CD30.CCR4)	CD30+ T-NHL	Relapse after high dose therapy and APSCT (ATLAS)	I	18	AEs, PFS, OS, survival of CAR in vivo	≥3	Active, not recruiting	NCT02663297
***CD37***									
	CAR-37 T cells	CD37+ R/R T-NHL	Mature T cell neoplasms R/R after 2 or more prior lines of therapy or Relapse after APSCT	I (FIH)	18	DLT, AE, OS, PFS, RR	≥18	Recruiting	NCT04136275
***CCR4***									
	Autologous CAR.CD30 T CELLS with CCR4 (ATLCAR.CD30.CCR4)	CD30+ CTCL	All cutaneous CD30+ T-NHL	I	59	AE, PFS, BOR, ORR, OS	≥18	Recruiting	NCT03602157
***TRBC1/2***									
	Autologous anti-TRBC1 CAR	R/R TRBC1+ T-NHL	PTCL, AITL, ALCL, T-ALL	I	9	CAR-T cell expansion and persistence ORR, DOR, OS, PFS	18–70	Recruiting	NCT04828174
	AUTO4; RQR8/aTRBC1 CAR T cells	R/R TRBC1+ T-NHL	PTCL, AITL, ALCL	I/II	200	Safety, CR+PR, AE, time to response	≥18	Recruiting	NCT03590574

R/R: Relapsed/refractory; T-NHL: T-Non-Hodgkin’s lymphoma; T-ALL: T-acute lymphoblastic leukemia; DFS: disease-free survival; PFS: progression-free survival; OS: overall survival; PD: progressive disease; SD: stable disease; DLT: dose-limiting toxicity; AE: adverse event; RP2D: recommended phase 2 dose; PK: pharmacokinetics; ORR: overall response rate; TTR: time to response; ASCT: allogeneic stem cell transplantation; APSCT: autologous peripheral stem cell transplantation; FIH: first-in-human; BOR: best objective response; CR: complete remission; DOR: duration of response; PR: partial response; MTD: maximal tolerated dose; DCR: disease control rate; RFS: relapse-free survival; EFS: event-free survival; 2-y: 2-year; AITL: angioimmunoblastic T-cell lymphoma; ALCL: anaplastic large cell lymphoma; ALK: anaplastic lymphoma kinase; ATLL: adult T-cell leukemia/lymphoma; CCR4: C-C chemokine receptor type 4; CTCL: cutaneous T-cell lymphoma; NK: natural killer; NOS: not otherwise specified; PTCL: peripheral T-cell lymphoma; TCR: T-cell receptor; TRBC: T-cell receptor β-chain constant domain.
